# Economic feasibility of interventions targeted at decreasing piglet perinatal and pre-weaning mortality across European countries

**DOI:** 10.1186/s40813-022-00266-x

**Published:** 2022-06-01

**Authors:** Anna H. Stygar, Ilias Chantziaras, Dominiek Maes, Vivi Aarestrup Moustsen, Dimitri De Meyer, Hélène Quesnel, Ilias Kyriazakis, Jarkko K. Niemi

**Affiliations:** 1 Natural Resources Institute Finland (Luke), Bioeconomy and Environment, Latokartanonkaari 9, 00790 Helsinki, Finland; 2 Unit of Porcine Health Management, Faculty of Veterinary Medicine, Ghent University, Salisburylaan 133, 9820 Merelbeke, Belgium; 3 SEGES Innovation P/S, Agro Food Park 15, 8200 Aarhus N, Denmark; 4 Vedanko BV, Knijffelingstraat 15, 8851 Koolskamp, Belgium; 5 PEGASE, INRAE, Institut Agro, 35590 Saint-Gilles, France; 6 Institute for Global Food Security, Queen’s University, Belfast, BT7 1NN UK; 7 Natural Resources Institute Finland (Luke), Bioeconomy and Environment, Kampusranta 9, 60320 Seinäjoki, Finland

**Keywords:** Neonatal mortality, Optimization, Welfare, Stillbirth, Human–animal interaction, Housing, Behavior

## Abstract

**Background:**

Piglet perinatal and pre-weaning mortality is a welfare problem causing economic losses in pig production. In this study, the effects of housing and management interventions on the economic result of sow enterprises representing six European countries (Belgium, Denmark, Finland, Germany, The Netherlands and Spain) were tested. Interventions concerned: (1) installing mechanical ventilation, (2) re-designing of the gestation unit, (3) drying and warming newborn piglets, (4) providing enrichment for gestating sows, including high-fiber dietary supplementation and point-source objects, and (5) music provision and backscratching of sows in the farrowing unit. A bio-economic model was used to determine the effects of interventions on economic outcomes during the nursery phase and to calculate a maximum cost of 1%-point reduction in perinatal and pre-weaning mortality, irrespective of the intervention type. Biological parameters were set according to previous observational and experimental studies. Interventions 1–4 were expected to decrease perinatal mortality, defined as stillbirths and deaths occurring within the first 48 h of postnatal life. Intervention 5 was expected to decrease pre-weaning mortality. Interventions increased fixed (1–3) and variable costs (3–5). We hypothesized that housing and management interventions would have a positive economic effect.

**Results:**

Piglet mortality can be decreased in various ways. Interventions concerning ventilation and re-designing of the gestation unit (1 and 2) were the most beneficial in countries with low housing costs and high perinatal mortality. Drying and warming newborn piglets (3) resulted in varying economic results, with the highest increase in profits obtained in a country with low labor costs and high litter size. Interventions providing sows with enrichment and human–animal interaction (4 and 5) were effective across varying conditions. Regardless of intervention type, policies aiming at 1%-point reduction in perinatal and pre-weaning mortality could cost from €0.2 to €0.5 (average €0.4) and from €0.4 to €0.5 (average €0.5) per piglet, respectively, depending on productions conditions.

**Conclusions:**

To decrease piglet mortality, farmers should consider low input interventions, such as those targeting appropriate behavior. Our results suggest that providing enrichment or increasing human–animal interaction pays off and brings positive economic result even when piglet mortality is marginally reduced.

## Background

Piglet perinatal and pre-weaning mortality is an economic, welfare and environmental concern. Piglets dying before or during farrowing, as well as before weaning can result in loss of revenues and extra production costs, reaching between €12 and €23 per litter reduction in returns [1]. Furthermore, piglet deaths may involve pain and/or suffering (mortality caused by chilling, starvation, injury, low birth weight or disease), which is considered a welfare issue [2, 3]. Decrease in production efficiency is also associated with higher environmental impact of pig production [4–7]. According to data on piglet mortality presented by studies from various European countries, one of five piglets will typically be stillborn or die within the first few days of life [8–10]. Piglets die from a wide variety of causes which are induced by the three-way interactions between the piglets, the sow and the environment [8]. Piglet mortality can be considered a production disease, which is defined as a disease originating from a complex interaction between the pathogen (where present), the animal and the environment where it is kept [11].

To date, several studies showed the economic benefits of various interventions aiming to improve the health of pigs. For example, improvements in biosecurity [12, 13], and vaccination of sows and piglets [13, 14] are cost-effective strategies in reducing impacts of production diseases in the pig industry. Furthermore, the reduction in pre-weaning mortality can lead to a substantial increase in profit obtained by sow herds [15]. However, so far, the effects of interventions have been tested on a limited number of farms, typically originating from a single country (e.g. [13]). Furthermore, economic analyses have concentrated mostly on interventions related with improving animal health, while little attention has been given to interventions aiming at reducing mortality by tackling other aspects of pig welfare, such as appropriate behavior (good human–animal interaction, enrichment material) or improved feeding. Previous studies, using data from several EU countries, identified different risk and protective factors related to piglet survival [16], as well as numerous management policies for providing good housing, feeding and appropriate behavior aiming to control piglet mortality [16–21]. However, the economic rationale for using these interventions has not yet been investigated.

Surveys concerning the attitudes of farmers to different preventive measures have indicated that producers viewed vaccination, reduced stocking density, adjustment to feed composition as well as enhanced monitoring, biosecurity, hygiene and additional enrichment as the most effective ways to control production diseases in pig livestock industry [22–24]. They ranked the improved profitability resulting from higher productivity as one of the most important factors motivating the implementation of measures to enhance disease prevention [25–27].

Across-country costs comparison shows that EU countries differ in terms of costs of pig production at farm level [28]. Moreover, there are substantial differences between countries in terms of farm performances expressed for example in the average number of piglets born alive, stillborn and weaned per litter [16]. Therefore, some health management policies which bring positive economic results in one country, may not necessarily be as economically beneficial in another country. Consequently, evidence on the financial impact of management modifications to reduce and to better control production diseases and decrease piglet mortality in pig systems across European countries is needed.

Different methods can be used to analyze economic consequences of interventions in herd management. The integration of economic and biological components within dynamic optimization models offers an opportunity to test the feasibility of potential management interventions and analyze economic attractiveness of production approaches aiming at decreasing piglet mortality. To date, Huirne et al. [29], Kristensen and Søllested [30, 31] as well as Niemi et al. [32] developed optimization models to study replacement decisions in farrowing herds. Each of these models contained parameters describing piglet mortality. Huirne et al. [29] tested the effect of decreased or increased pre-weaning mortality on economic outcomes per sow, but in the economic model deaths before or during farrowing were not defined as separate parameter. Also, Kristensen and Søllested [30, 31] modeled piglet mortality based on total number of piglets born assuming that all deaths will occur within the first week after birth. However, the main focus of this study was to construct a decision support tool for pig farmers [33], and detailed analyses of the influence of mortality rate on economic results obtained at the farm level in Denmark were not provided. Finally, Niemi et al. [32] analyzed different reasons of piglet mortality (caused by postpartum dysgalactia syndrome (PDS) or locomotory disorder) and their influence on the economic result of piglet production.

Piglet mortality can be influenced by a variety of conditions, and there is a need to consider interventions aiming at reducing it. Some of these interventions may be associated with financial consequences, which were evaluated in this paper. To the best of our knowledge, the present study is the first attempt to quantify the cost for piglet mortality reduction across European countries. The aim of this study was to assess the financial consequences of five interventions to reduce piglet mortality. Studied scenarios were selected among those examined within the EU FP7 PROHEALTH consortium and concerned factors related to housing (ventilation and interior) as well as to feeding and appropriate behavior (assistance, enrichment, animal-friendly handling). The financial consequences refer both to the benefits obtained because of intervention and to the costs of implementing an intervention itself. Our hypothesis was that interventions aiming at improving animal welfare would increase the financial value of a sow space unit.

## Material and methods

### Model structure

Different methods can be used to analyze economic consequences of interventions in herd management [34]. Given that interventions related to piglet perinatal and pre-weaning mortality might influence sow replacement decision, methods suited for determining optimal livestock replacement decisions, are required to be applied. Dynamic programming meets this requirement by optimizing sequential decision-making problems under uncertainty. A bio-economic model characterizing the most important productive traits and integrating the biological and economic consequences of farmer’s activities in farrowing sow production, was used for analyses. In this study, the framework of the stochastic dynamic optimization model of the farrowing unit described by Niemi et al. [32] was modified to represent production conditions of six EU countries (Belgium, Denmark, Finland, Germany, The Netherlands and Spain) and cases presented in subsequent sections. The basic structure of the model is presented in this section, the next sections describe parameter values used in the modeling for basic scenario and all tested interventions.

The objective of the model was to maximize the net return on a sow space unit by optimizing the sow replacement policy. Sow space unit referred to the housing capacity that a sow and her piglets required during the production cycle. The model characterized cash flows, production and health parameters related to the sow and its offspring and interactions between these. One of the main effects taken into account by the model was the influence of production parameters on the lifespan of the sow and the offspring it can produce. Hence, the model was used as a device to quantify financial impacts of different management modifications targeting the sow and piglets.

The optimization problem was formulated by the Bellman equation [35]:

$$\begin{aligned} {V}_{t}\left({\mathbf{x}}_{\mathbf{t}}\right)=&\underset{{u}_{t}}{max}\left\{{R}_{t,sow}\left({\mathbf{x}}_{\mathbf{t}},{u}_{t}\right)+\beta E\left({V}_{t+1}\left({\mathbf{x}}_{\mathbf{t}+1}\right)\right)\right\},\\& t=1,...,\infty \quad \mathrm{and} \quad\mathrm{where}\end{aligned}$$

$${\mathbf{x}}_{\mathbf{t}}=\left\{{x}_{t,prices},{x}_{t,disease},{x}_{t,parity},{x}_{t,litter}\right\}$$

$${\text{Subject to}}:x_{t + 1,litter} = g(x_{t,parity} ,x_{t,litter} ,u_{t} ,\varepsilon_{y} )$$

$$x_{t,disease} = \Pr_{disease} (x_{t,yield} ,x_{t,parity} )$$

$$x_{t + 1,parity} = q(x_{t,disease} ,x_{t,parity} ,x_{t,litter} ,u_{t} )$$

$${\mathbf{x}}_{\mathbf{t}} \,\text{and} {V}_{\infty }({\mathbf{x}}_{\mathbf{\infty }})$$

are given where *t* is a time index representing farrowings elapsed since the insertion of a sow into the production unit; ${\mathbf{x}}_{\mathbf{t}}$ is the state vector, ${V}_{t}\left({\mathbf{x}}_{\mathbf{t}}\right)$ is the value function (i.e., the maximized value of a sow space unit as a function of the state variables) in time period *t*; $R_{t,sow}$ is a one-period returns function for the time period *t*; $u_{t}$ is a binary control variable taking values [0,1] for both replacement (0 = keep the current sow, 1 = replace the current sow with a new gilt) and intervention (however, the intervention is pre-defined and assumed to be applied constantly throughout the lifespan of the sow whereas the replacement decision varies by parity, litter size and disease); $\beta$ is a discount factor; $E(.)$ is an expectations operator applied on the term inside brackets; $V_{t + 1} (x_{t + 1} )$ is a value function at period *t* + 1; *g* and *q* are transition equations representing the development of litter size and parity number from production cycle to production cycle; and $\Pr_{disease}$ is an equation representing the occurrence of disease (modeled for PDS, locomotory disorders and any other disease) and its impacts in the model. The state vector ${\mathbf{x}}_{\mathbf{t}}$ consists of four variables identified by the subscripts. First variable, $x_{t,prices}$, represents country-specific prices related to production (e.g. feed, piglets, cost of replacement, cost of intervention). The second variable,$x_{t,disease}$, indicates the presence of disease in the sow or in the piglets and is used to quantify the impact of disease on them. The third and fourth variables describe the total number of piglets born in a given parity $x_{t,litter}$ and the number of parity $x_{t,parity}$.

A sow switches from one discrete production state to another (e.g. from one parity to another) according to the transition equations, the values of which depend solely on the current state of nature (litter size, parity number, disease status) and the decision made (the control variable). Price parameters affect the transitions indirectly because they influence the optimal decisions. Uncertainty about the development of economic returns is represented in the model by the probability of observing a disease in a sow (PDS, leg disorders or other disorders) during the current parity, variation related to litter size between successive parities (assumed variation described by $\varepsilon_{y}$), and the mean of piglet mortality and the variation of piglet mortality, and the likelihood of culling (e.g. disease-related culling) a sow.

One-period returns depended on revenue from selling piglets and on expenses, such as feed costs, insemination, sow replacement costs, labor, veterinary services. Fixed costs of production, *e.g.* housing costs, which influenced the economic result but not the optimal pattern concerning replacement, were also included in the model.

In the bio-economic model, perinatal mortality was defined similarly to the study of Pandolfi et al. [8] and comprised of mummified piglets, still-born piglets, and piglets born alive which died within the first 48 h of postnatal life. Pre-weaning mortality was calculated based on all piglets dying from two days of postnatal life to weaning. The perinatal ($PeriMor$) and pre-weaning ($PreMor$) mortality rates were obtained using following equations:

$$PeriMor=\left(0.072+0.011\mathrm{ln}\left({x}_{t,parity}\right)+0.103\mathrm{Pr}\left({x}_{t,PPDS}|{x}_{t,parity}=1\right)+0.019\mathrm{Pr}\left({x}_{t,PPDS}|{x}_{t,parity}>1\right)+0.073\mathrm{Pr}\left({x}_{t,legs}|{x}_{t,parity}=1\right)+0.094 \mathrm{Pr}\left({x}_{t,other}\right)\right)*{Cor}_{1}$$

$$PreMor=\left(0.096+0.018\mathrm{ln}\left({x}_{t,parity}\right)\right)*{Cor}_{2}$$

Perinatal and pre-weaning mortality for piglets depended on the litter size in a given parity ( ${x}_{t,parity}$), probabilities of sow suffering from leg disorder in the first parity $\mathrm{Pr}\left({x}_{t,legs}|{x}_{t,parity}=1\right)$, PDS in the first $\mathrm{Pr}\left({x}_{t,PPDS}|{x}_{t,parity}=1\right)$ and following parities $0.019\mathrm{Pr}\left({x}_{t,PPDS}|{x}_{t,parity}>1\right)$ as well as probability of sow suffering from some other diseases$0.073\mathrm{Pr}\left({x}_{t,legs}|{x}_{t,parity}=1\right)$. Both equations were adjusted by country correction factor (${Cor}_{1} , {Cor}_{2}$) to represent average mortality rates in a given country.

A policy iteration method was used to solve the replacement problem. Further details on the bio-economic model are provided by Niemi et al. [32]. The model was programmed in Matlab 2014b [36].

### Parametrizing the model

The model was defined to represent parameters specific for six of the countries participating in the PROHEALTH consortium, namely Belgium, Denmark, Finland, Germany, The Netherlands and Spain [16, 37]. Due to data anonymity requirements, countries were coded as A, B, C, D, E and F; the letter order is not consistent with the country order mentioned above. For each country, specific parameters representing prices ($x_{t,prices}$) and production parameters were determined. Hence, the parameters reflected differences between countries in price and production parameters. The most important production parameter values, such as litter size of a gilt, perinatal and pre-weaning mortality were based on results presented by Chantziaras et al. [16] and Niemi et al. [32]. The production parameter data were obtained from datasets recorded between 2014 and 2015 and originating from 131 farms across Europe. Price parameters, representing average values for year 2017, were set according to the report of Hoste [28]. Labor estimates and the pricing model for weaners were set based on information obtained through personal communication with the members of the PROHEALTH consortium. The production and price parameters are summarized in Table 1. Studied examples varied in farm performance, costs of production and revenues. Country A was characterized by low costs of production (except feeding costs), contrary to country F, which was characterized by high costs of production, except feeding costs. Sows in country D achieved the highest litter size, but also the highest stillbirth and pre-weaning mortality among all countries. Country B was characterized by exceptionally high labor costs and low piglet price. On the other hand, country E had the highest price of piglets. Country C had moderate values regarding farm performance, costs and revenues.

**Table 1:** Costs, revenues and performance parameters used in the bio-economic model for six countries

Bio-economic parameters	Country^1^					
	A	B	C	D	E	F
**Economic parameters**						
Gestation feed (€/1000 MJ NE)	20.61	19.12	20.39	18.27	18.69	17.42
Lactation feed (€/1000 MJ NE)	25.07	23.26	24.81	22.22	22.74	21.19
Piglet feed (€/1000 MJ NE)	46.55	43.19	46.07	41.27	42.23	39.35
Price of labor (€/h)	14	25	16	22	18	18
Labor per sow/year (h)	8.1	7.5	10.7	11	12.0	10.4
Labor per litter (h/litter)	1.69	1.67	1.67	2	1.67	1.67
Labor per litter (h/day)	0.03	0.03	0.03	0.06	0.03	0.03
Labor per weaner (min/day)	0.34	0.40	0.40	0.13	0.40	0.40
Labor per insemination (h)	0.5	0.5	0.5	0.5	0.5	0.5
Fixed cost of housing (€/m^2^)^2^	118	240	246	257	284	351
Price of gilt (€/gilt)	200	265	310	226	275	350
Price of insemination dose (€/dose)	3.2	3	3.6	4	2.7	5
Value of culled sow (€/sow)	190	124	170	152	165	108
Sale price of weaner, (€/weaner)^3^	53	35.2	45	44	59	55
**Farm performance**^**4**^						
Number of liveborn piglets per litter	12.7	14.4	14.4	16.1	13.6	11.6
Stillbirths	8.7	7.4	8.1	9.5	7.2	7.2
Pre-weaning mortality %	11.9	13.5	13.5	15.2	12.8	10.7

^1^The values have been obtained from literature [16, 28, 32] as well as personal communication with members of the PROHEALTH consortium. The six countries were randomly coded as A, B, C, D, E and F due to anonymity requirements

^2^Including country differences for requirements concerning m^2^ for piglets and sows, farm size, quality of the building (lifetime) and the ratio of a number of places (*e.g.* countries with a high sow performance need more piglet and fattening places per sow than countries with a lower performance). Because the cost of housing capacity is a time-constant factor, these costs did not influence the optimal timing of replacement

^3^For country C, D, E and F the price was estimated for 30 kg piglet, for country A the price was for 20 kg piglet, for country B the price was for 25 kg piglet

^4^Calculated based on data collected from farms across Europe [16, 32]

For all countries, a discount factor (6% annual interest rate), a maintenance cost of housing (1%) and overhead costs (4% for every cost included in the model) were assumed [32].

The effects of interventions on financial performance of the farm were modeled as follows. Firstly, results for the baseline scenario (i.e. model run without any intervention assumed) were produced for all countries. Secondly, model parameters were adjusted to take into account the effects of an intervention, and the model was re-run with these parameter values and the assumption that a respective intervention was applied. Thirdly, an expected value of sow space unit, produced by the model, with the intervention in place was compared with the value obtained for the baseline scenario to obtain the financial impact of the intervention. Based on the results of underlying supranational analyses conducted by Chantziaras et al. [16], it was assumed that the effect of intervention on production parameters was similar in each country.

### Cost of 1%-point reduction in mortality rates

In order to determine cost of 1%-point reduction in mortality rates, for each considered country, the model inputs ($PeriMor$, $PreMor$) were varied independently to represent 1%-point reduction in perinatal and pre-weaning mortality. Other model parameters remained unchanged. Net present value of the sow space unit obtained in “Baseline scenario” was later subtracted from net present value obtained under the assumption of 1%-point reduction in perinatal and pre-weaning mortality. This approach allowed us to generalize the results and provide general cost threshold applicable for any intervention type aiming to decrease piglet mortality (perinatal and pre-weaning).

### Baseline scenario

The baseline scenario was produced by running the model with the baseline parameter values (see Table 1). In the baseline scenario, the modeled farm was following standard health management policies. The standard health management policies were defined for farms which were applying only a basic assistance during farrowing, namely providing assistance for sows with dystocia and a supplementary heating in creep area (1 lamp at birth). Furthermore, it was assumed that in the baseline scenario, gestating sows were kept in buildings with interior designs older than 12.5 years and equipped with natural ventilation. Finally, the basic settings assumed that gestating sows were provided with enrichment material to meet the requirements of EU Council Directive 2008/120/EC and Commission Recommendation (EU) 2016/336 of 8 March 2016 (in the analyzed baseline scenario point-source objects were not available).

### Interventions

Interventions were selected among those examined within the PROHEALTH project [16, 18–21]. Interventions fell into three classes: (1) associated with improvements in the housing environment, (2) related to management or (3) affecting both housing and management. The interventions were selected to cover varied welfare principles (good housing, feeding and appropriate behavior), as well as cost of interventions (fixed costs of housing and variable costs of feeding as well as labor). Assumptions concerning interventions, their effects on productivity and approximated additional costs are summarized in Table 2. All tested interventions were assumed to be implemented independently.

**Table 2:** Basic assumptions on productivity and additional costs for management and housing interventions tested in the bio-economic model

Intervention name^ 1^	Description of an intervention	Expected effect on mortality reduction (%)		Cost of intervention
		Perinatal mortality	Pre-weaning mortality	
**Housing interventions**				
(1) Ventilation	Mechanical ventilation in gestation unit	3.8% [16]		Fixed costs up by 1% (own calculations)
(2) Interior	Interior design of gestation unit renewed every 12.5 years	2.6% [16, 18]		Fixed costs up by 5% (own calculations and [38])
**Management and housing intervention**				
(3) Assistance	Drying and placing piglets close to udder, 3 heating lamps per litter	2.4% [18]		Labor input and fixed costs up by 10 and 0.25%, respectively (own calculations and [39])
**Management interventions**				
(4) Enrichment	Oak attached to a chain (3 per pen), straw pellets supplementation	4% [20]		Cost of feed up by 1%, cost of enrichment set at €1.8/sow (own calculations and [40])
(5) Animal-friendly handling	Sows experienced music (06.00–18.00) and backscratching (15 s/sow/d) during farrowing and lactation period		3.3% [19, 21]	Additional 9 min labor per litter [41]

^1^All interventions were assumed to be implemented independently

### Profitability requirements

For each intervention, as well as each country, input variables representing costs and mortality rates were varied independently to determine how the outcome indicator (net present value of a sow space unit) was changing in each situation. Profitability requirements for cost were obtained by increasing the costs of interventions, with other input parameters held constant, to the value when obtained profit from sow space unit was lower than in the baseline scenario. The maximum cost not resulting in the financial losses was selected as a cost threshold.

A similar approach was assumed for the profitability requirements concerning change in mortality rate. For each intervention, effect of intervention on mortality rate was systematically decreased until net present value of a sow space unit was equal to the baseline scenario. Profitability requirement for effect on mortality rates was reported as the maximum decrease resulting in a positive change in the farm’s economic result.

## Results

### Basic scenario

The expected value of the sow space unit, defined as the housing capacity that a sow and her piglets required during the production cycle, differed between countries (Table 3). The highest net return for sow space unit was obtained for country A characterized by low labor, housing and replacement costs and high piglet price. The lowest value of space unit (which was negative when including high fixed housing costs) was obtained for country D, characterized by high labor and housing costs and low replacement and piglet prices.

**Table 3:** Economic outcomes obtained from bio-economic model by pig farrowing farms in six EU countries

Economic outputs^ 1^	Country^1, 2^					
	A	B	C	D	E	F
Value of sow space unit (without fixed costs) in €	7437	3365	6016	1972	7224	4582
Value of sow space unit (with fixed costs^3^) in €	6157	501	3100	-1245	3920	795
Cost threshold for 1%-point reduction in perinatal mortality, €/sow space unit, (€/piglet)	111 (0.4)	126 (0.4)	197 (0.5)	96 (0.3)	71 (0.2)	142 (0.4)
Cost threshold for 1%-point reduction in pre-weaning mortality, €/sow space unit (€/piglet)	142 (0.5)	143 (0.5)	165 (0.4)	140 (0.4)	180 (0.5)	157 (0.5)

^1^ The economic outputs are related to value of sow space unit and the maximum costs of 1%-point reduction in perinatal and pre-weaning mortality. The negative value of sow space unit for country D denotes that under assumed economic and production parameters, considering fixed costs of housing, piglet production was not profitable

^2^Countries were randomly coded as A, B, C, D, F due to anonymity requirements

^3^Discounted revenues minus all discounted costs including *e.g.* housing and insurance

### Costs of 1%-point reduction in piglet mortality

Based on the results, irrespective to the intervention type, economically feasible policies aiming at 1%-point reduction in perinatal mortality can cost, on average, up to €0.4 per piglet (from up to €0.2 per piglet in country E to as much as up to €0.5 per piglet in country C—Table 3). These results correspond to €71 and €197 extra costs per sow space unit per year, respectively. Regarding economically feasible policies aiming at 1%-point reduction in pre-weaning mortality, the cost thresholds for both piglet and sow space unit were characterized with smaller variation between analyzed countries. To reduce pre-weaning mortality by 1%-point, farmers could afford to pay on average €0.5 per piglet (from € 0.4 to 0.5 per piglet, which corresponds to €140 to 180 per sow space unit—Table 3).

### Interventions

**Fig. 1:**
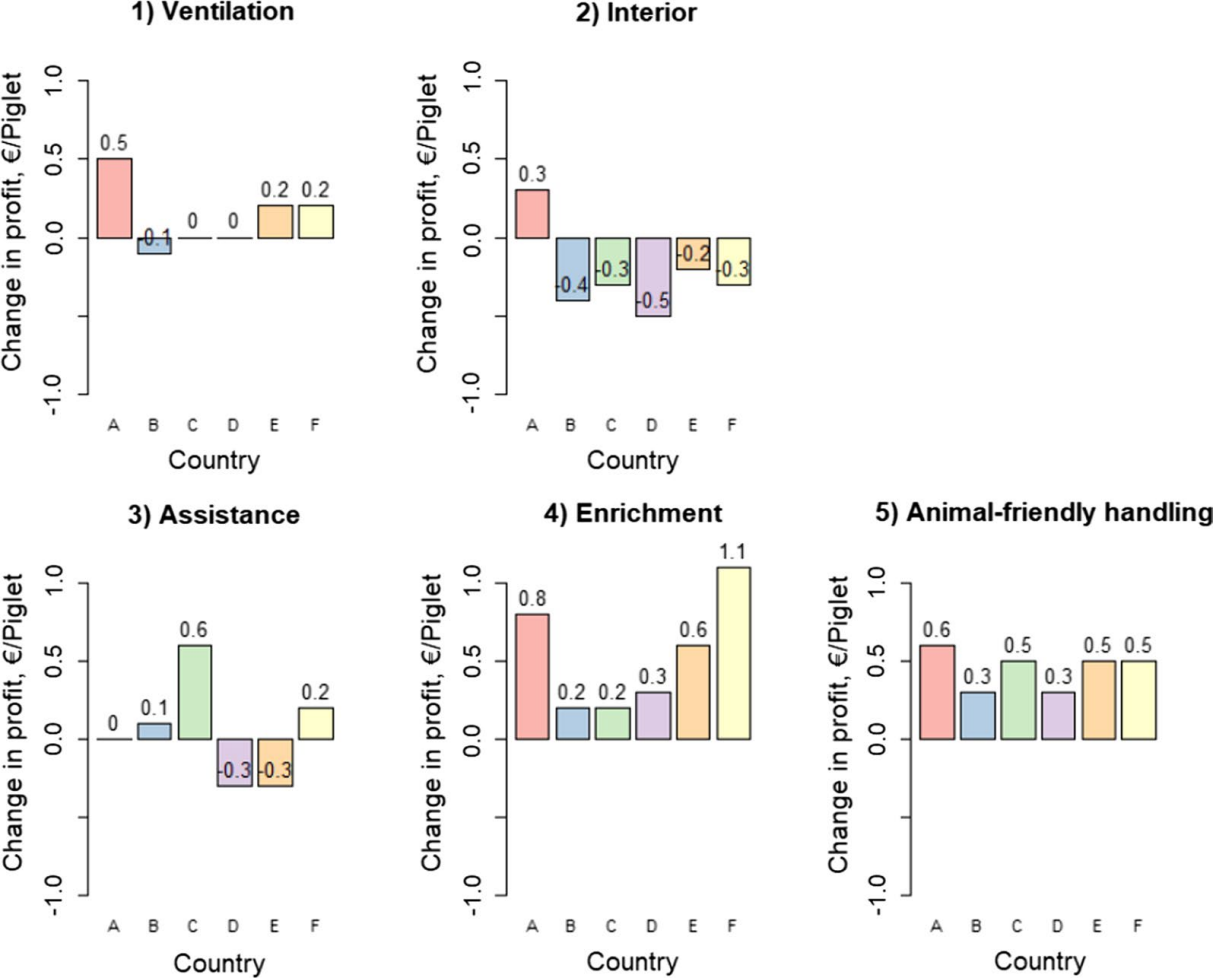
Effect of intervention on expected profit per piglet compared to baseline scenario in six EU countries. Interventions concerned:** 1** Installing mechanical ventilation in a gestation unit,** 2** Re-designing of a gestation unit,** 3** Drying and additional heat source** 4** Providing enrichment for gestating sows and ** 5** Music and backscratching of sows in the farrowing unit

The change of profit for all analyzed interventions is presented in Fig. 1. For the assumed costs of interventions, installing mechanical ventilation in a gestation unit, resulted in positive effect on economic result in country A, E and F. Re-designing of a gestation unit interior was economically feasible in country A only. Providing assistance (regular help to piglets and several sources of supplementary heating) had a negative impact on the economic result in country D and E, did not change obtained profits by farmers in country A and resulted in positive changes to economic results in countries B, C and F. Providing enrichment for gestating sows (wood, chain and straw pellets) and animal friendly handling (music and backscratching of sows in a farrowing unit) were the most beneficial interventions across analyzed countries. Under conditions prevailing in the studied scenarios, both interventions resulted in positive change in profit obtained per piglet in all six EU countries.

### Profitability requirements for tested interventions

Results for minimum profitability requirements, both for cost and mortality parameters, are presented in Table 4. According to the basic assumptions, installing mechanical ventilation were estimated to increase fixed costs by 1% (Table 2). However, with unchanged production benefits concerning reduction in piglet mortality, the farmers from countries A, E and F could invest more before this intervention became economically non-viable (2% up to 10% increase in fixed costs). This intervention also had wide profitability range regarding production performance, especially for country A. For example, with unchanged cost of intervention for country A, installing mechanical ventilation could have marginal effect on perinatal mortality, and still remain profitable. Regarding interior intervention, re-designing the farrowing unit was expected to increase fixed costs by 5%. However, assumed costs in relation to the effect on production were too high, resulting in decreased value of sow space unit in most analyzed countries.

**Table 4:** Minimum profitability conditions for interventions in six EU countries

Intervention	Input variable ^1^	Basic assumptions for interventions	Conditions for minimum profitability^2^					
			A	B	C	D	E	F
**Housing intervention**								
(1) Ventilation	Fixed costs increase (%)	1	10	NA	1	NA	3	2
	Perinatal mortality (%)	3.8	0.4	NA	3.0	NA	1.1	1.9
(2) Interior	Fixed costs increase (%)	5	17	NA	NA	NA	NA	NA
	Perinatal mortality (%)	2.6	1.3	NA	NA	NA	NA	NA
**Management and housing intervention**								
(3) Assistance	Labor input increase (%)	10	10	11	15	NA	NA	11
	Perinatal mortality (%)	2.4	2.4	2.2	1.5	NA	NA	1.9
**Management intervention**								
(4) Enrichment	Cost of enrichment material/sow (€)	1.8	10	5	6	7	10	14
	Perinatal mortality (%)	4	1.6	3.2	2.8	2.4	1.6	1.2
(5) Animal-friendly handling	Additional labor per litter (minutes/litter)	9	23	13	23	12	22	19
	Pre-weaning mortality (%)	3.3	1.3	2.3	1.3	2.3	1.6	1.6

^1^Input variables were independently varied to determine the change in mortality rate or the change in costs which would have resulted in the value of sow space unit being equal to the value obtained in the baseline scenario

^2^Six countries were randomly coded as A, B, C, D, E and F due to anonymity requirements

NA- not applicable, profitability requirements were not estimated for interventions with negative economic result

Regarding factors related to animal management, assistance scenario resulted in noticeable economic benefits, but only for selected production conditions (country B, C, F). Moreover, for those countries, range for both production and cost parameters remained narrow. For example, for countries B and F the economically viable intervention permitted only 1%-point increase in the labor input compared to baseline settings.

Providing enrichment for gestating sows was found to be a profitable intervention in all six countries. Assuming unchanged costs, this intervention would remain profitable even if piglet perinatal mortality would be reduced by only 1.2%-point in country F (compared to initially assumed 4%-point reduction). However, for selected production conditions, the reduction in mortality incidents would need to be more substantial (approximately 3% for countries B and C). With unchanged effect on production parameters, the intervention was expected to be economically viable when enrichment materials cost between €5 and €14.

For animal-friendly handling, the labor costs thresholds could increase for all countries (from 12 min up to even 23 min of additional labor input per litter) compared to basic scenario (which assumed 9 additional minutes). The intervention on animal-friendly handling was set to decrease piglet weaning mortality in “Baseline scenario” by 3.3%. However, for all six countries the effect on production could be reduced substantially before intervention became unprofitable. The performance threshold was set at the highest level for countries B and D (2.3%-point reduction) and the lowest level for countries A and C (1.3%-point reduction).

## Discussion

The financial impacts of implementing different animal and housing management interventions aiming to decrease perinatal and pre-weaning piglet mortality were investigated by using data from different sow farms in six European countries. In order to represent the farrowing unit, a stochastic dynamic model was implemented for various European production conditions and used to calculate economic results. We expected that interventions aiming to improve animal welfare would result in increased economic result from a sow space unit across varying production conditions.

Our results indicated that some of the examined interventions appeared to be profitable for all pig farmers of the six analyzed countries (supplying enrichment for gestating sows, and assuring animal friendly handling), while the others were economically viable for only selected conditions (installing ventilation and re-design of gestation unit, assistance for piglets). Housing interventions were the most beneficial for farmers in countries with lowest housing costs. Management interventions, such as increased human–animal interaction as well as provision of enrichment, seemed to be effective across all countries. Interventions involving both housing and management seem to yield varying results, with the highest increase in profits obtained in country with low labor costs and high litter size. Overall, interventions aiming to reduce 1%-point in the perinatal and pre-weaning mortality are economically feasible when costing up to €0.4 and €0.5 per piglet, respectively. Finally, profitability analyses suggested that providing enrichment or increasing human–animal interaction were bringing positive economic results, even with reduced efficiency of intervention. For example, perinatal mortality could be reduced in enrichment intervention by 1.2% instead of the originally assumed 4% to remain profitable for country F. The effect that animal-friendly intervention has on mortality rate could be decreased by almost two thirds and still it would have remained profitable for countries A and C.

The results from the PROHEALTH consortium [16, 18–21] served as the starting point to explore the economic rationale of management procedures to combat piglets mortality. An interested reader can find the discussion on the values of biological parameters (perinatal and pre-weaning mortality) in publications analyzing following conditions: ventilation and housing [16], piglet assistance [18], enrichment [20] and animal-friendly handling [19]. However, in this study the discussion will be limited to the socio-economic aspects of analyzed interventions.

### The economic efficiency of interventions

To the best of our knowledge, this study is the first attempt to determine the economic value of interventions to reduce piglet mortality across European countries. Therefore, the comparison of results with previous studies is challenging. As far as general trends are concerned, our results seem to be in agreement with an earlier study suggesting that improved pig welfare can be achieved even with a modest increase in cost [42].

In this study, we considered interventions related to housing and animal management. The interventions related to housing conditions mainly affected fixed housing costs. Regarding interventions aiming at animal management, these were affecting variable costs (enrichment, animal friendly handling). Providing piglets with assistance concerned both housing and animal management, therefore affected both variable and fixed cost. Unsurprisingly, interventions related to both cost categories resulted in higher variation between countries in the expected return per piglet compared with intervention affecting only one cost category.

Regarding the profitability requirements, our results are in agreement with the recent study concerning the cost-effectiveness analysis of measures to reduce tail biting in fattening pigs [43]. According to Niemi et al. [43] interventions which were considered the least expensive to apply (e.g. such as provision of point-source enrichment objects) or provided wider production benefits (e.g. improvement in ventilation), became profitable at a lower level of efficacy than measures which were considered the most expensive to apply (e.g. an increase in space allowance due to building refurbishment) and affect fewer production parameters. A similar trend regarding low-cost interventions can be noticed also based on our analyses. Providing enrichment material for gestating sows was profitable across the examined countries. Regarding ventilation, our modelling approach assumed rather limited influence on production parameters (perinatal mortality rate). However, even with a narrow production benefit, installing mechanical ventilation was profitable or did not substantially affect the value of sow space unit in most of analyzed countries. Recent studies suggest that installing mechanical ventilation can reduce antimicrobial consumption [44]. Therefore, potential financial benefits for farmers installing mechanical ventilation, due to e.g., decreased medication costs, might be greater than indicated by our results.

### Interventions related to housing conditions

The analyzed interventions concerned installing mechanical ventilation and re-designing the gestation unit. These two interventions, aimed at decreasing perinatal mortality, affected fixed housing costs. The costs of buildings renovation might differ depending on building types, labor prices, climatic conditions and other factors, and hence vary from farm to farm. The countries considered in our study represented the diversity of production systems in different climatic conditions (boreal, continental, Atlantic, Mediterranean). The result suggests that countries with the lowest costs of housing and labor, but high rate of perinatal mortality can benefit the most from housing related interventions aiming to improve animal welfare. In the study of Chantziaras et al. [16] the age of facilities was associated with standards of management, housing and biosecurity. However, refurbishment to achieve those standards might not always be possible. In some cases, building new facilities according to most welfare-friendly standards (e.g. an increase space allowance, an increase capacity for bedding and nesting material [45]) might be a preferred solution. In the meantime, farms with old buildings should consider interventions related to animal management, which in a short term might be economically viable actions for reducing piglet mortality.

The study of Chantziaras et al. [16], used to set the model’s basic parameters, did not report more detailed data concerning type of used equipment in refurbished buildings. Therefore, the results shall be interpreted with caution, as some changes to the interior design might be more profitable than others. For example, the designed (high welfare) farrowing pens were proven to economically over-perform systems based on ordinary pens and crates when piglet survival rate was adjusted by increased space allowance, extra substrate and modified pen heating [46].

### Interventions related to animal management or combination of animal management and housing

Two animal management interventions, namely enrichment and animal friendly handling, were investigated. These interventions concerned providing enrichment for gestating sows, including high-fiber dietary supplementation and point-source objects to decrease perinatal mortality as well as music and backscratching of sows in the farrowing unit to reduce pre-weaning mortality. Furthermore, in the assistance scenario focusing on drying and warming newborn piglets, we analyzed combined effect of housing and management intervention on perinatal mortality. Regarding management interventions, our results indicated that the provision of point-source objects is financially viable for reducing perinatal mortality. Similar results were obtained in a previous study on cost-effective measures to reduce tail-biting in fattening pigs [43], mostly due to the relatively low prices of intervention. Even though, in our study, enrichment consisted of both high-fiber diet and provision of wood attached to a chain, the measure was quite inexpensive to be adopted across different production conditions. Regarding increasing human–animal interaction, our results suggest that actions targeting appropriate behavior can bring economic benefits across different production conditions in the EU. Any similar economic results concerning human–animal interaction were not identified in the literature. Yet, there is evidence that increased interaction between the pigs and the farmer can have positive effect on animal welfare and farm productivity [47]. Kirkden et al. [48] reviewed different management procedures to improve piglet survival and pointed out that labor intensive interventions might result in a net economic benefit even in countries where labor is costly. This was also confirmed in our study. For example, an additional 9-min labor input per litter in the scenario assuming animal-friendly handling was associated with an increased profit per piglet for countries with the highest (country B) as well as the lowest (country A) labor price. Economic results in the scenario with additional assistance for piglets varied substantially between analyzed countries. This intervention assumed the lowest change in perinatal mortality with quite substantial financial inputs (labor and increase in fixed costs). According to the literature [49], the cost of drying piglets has not been appraised previously.

### Incentivizing animal welfare interventions

The prospect of improving the image of pig production and the economic incentive motivate many farmers to participate in actions aiming to improve animal welfare [50]. However, as recently discussed in the context of reducing aggression in fattening pigs, farmers should not be considered a homogenous group concerning the adoption of animal welfare innovations [51]. For example, some producers might remain cautious about large-scale investments in welfare friendly solutions [52]. Therefore, interventions should be targeted, depending on farmer preferences regarding changes in production as well as their willingness to pay for different management interventions.

As demonstrated in this study, there are several cost-effective ways to decrease piglet mortality. Hence, similar to the conclusion of Peden et al. [51], further efforts should be concentrated on promoting these interventions to practice through increase science-farmer dialogue.

The current study examined selected interventions and provided insights on the economic rationale to reduce piglet mortality through selected changes in farm productivity. However, besides changes in piglet mortality, there may be also other benefits associated with interventions, which could incentivize farmers. Recent studies suggested that there is a connection between housing conditions and antimicrobial consumption (e.g. [53]) and changes in housing and management might in the long run decrease antimicrobial consumption (e.g. [44, 54]). Furthermore, by improving animal welfare and branding the welfare improvements (e.g. [55]), a price premium may be obtained for delivering higher-value products to the consumers. The two examples of additional benefits may suggest that even costly interventions could pay off for pig farmers in a longer time span. However, to estimate the effects of such additional benefits, more evidence on the potential effects of various interventions concerning housing, feeding, health and behavior on animal welfare and productivity, demonstrated on the population level data, is needed. In addition, quite little is still known about consumer willingness to pay for policies related to reduction in prevalence of specific production diseases [56].

### Study limitations

The model parameters were specified to estimate cost-effectiveness of various interventions combating piglet mortality across Europe. In order to provide conclusions for several European countries, initial parameters for modeling mortality rates and effects of interventions were derived from a cross-sectional study of Chantziaras et al. [16] and research conducted within the PROHEALTH consortium [18–21]. On the one hand, one may consider that the associations derived from an observational study cannot be readily extrapolated in casual relationships and should be confirmed with experimental research. On the other hand, as seen on the example of cost–benefit analyses of tail-biting lesions [43], parametrizing a bio-economic model using information from previously published experimental studies may be challenging. The challenge in parameterization is related with between-experiment variation in the efficacy of preventive measures.

In this study, the bio-economic model was parameterized to represent general country conditions. To provide decision support for individual farms, farm-specific data would need to be obtained. Production conditions, e.g. due to different biosecurity levels, on pig farms are not identical [57]. Therefore, specific interventions might not be equally effective between farms. Furthermore, as noticed by Niemi et al. [43] appropriate targeting of the measures is essential for their profitability because an intervention is not automatically always effective, and the selection of an intervention must be solution-oriented. For this reason, analyzing herd productivity and management as well as conducting small management experiments, involving part of the herd, might be advisable before adjusting whole management procedures. Monitoring tools able to assess an effect of small experimental changes, based on sensor date, during normal production cycle on commercial dairy farms [58, 59] and pig farms [60] were developed. However, the pig industry is behind dairy regarding sensor availability [61]. Therefore, the potential of utilization of those monitoring tools on commercial pig farms still needs to be explored.

This study covers interventions targeted to reduce piglet mortality which were selected based on the analyses carried out within the PROHEALTH project. Though, some other possible interventions could be considered in the future research. For instance, in recent years substantial scientific effort was made to develop various sensor technologies with potential to inform farmer about animal welfare [62]. However, the commercial availability and validation rate of such tools remain low [61]. This uncertainty regarding sensor performance, as well as uncertainty concerning potential benefits in decision support play an important role in farmers’ investment decisions [63]. Results obtained from bio-economic modeling could fill the knowledge gap regarding technological development and find practical use in evaluating the merits of various sensor systems for farm management.

The modeling approach used in this study is flexible but provides opportunities also for more rigorous analyses. The sensitivity of obtained solutions (sow replacement policies) can be tested by varying model parameters. Consequently, sensitivity analyses can be used to test, for example, how different values of a price premium could affect obtained results. Future scientific efforts should concentrate on combining the possible additional effects of investments and exploring in detail the economic trade-offs associated with the adaptation of different interventions to reduce piglet mortality.

## Conclusions

In this study, the influence of modifications targeting management and housing on the economic result of sow enterprises across EU countries were analyzed. Four of our interventions investigated the effect of improvements on piglet perinatal mortality, one intervention concerned improvement in piglet pre-weaning mortality. According to obtained results, different types of interventions can be used to decrease piglet mortality across Europe. We identified the economic consequences of such interventions and conclude that interventions aiming at enhancing appropriate behavior, such as providing enrichment or animal friendly handling were found economically viable for various production conditions. The profitability analyses indicated that farmers should pay special attention to low-cost interventions which become economically viable even at low level of efficiency. Investing in improving animal welfare does not necessarily require large capital investments on the farm. Our analyses showed that costs of interventions resulting in 1%-point reduction in perinatal and pre-weaning piglet mortality were reaching depending on the country from €0.2 to €0.5 (average value €0.4) and from €0.4 to €0.5 (average value €0.5) per piglet, respectively.

## Data Availability

Not applicable.
